# Development of Pasta Products with Nonconventional Ingredients and Their Effect on Selected Quality Characteristics: A Brief Overview

**DOI:** 10.1155/2019/6750726

**Published:** 2019-11-27

**Authors:** R. A. T. Nilusha, J. M. J. K. Jayasinghe, O. D. A. N. Perera, P. I. P. Perera

**Affiliations:** ^1^Department of Food Science and Technology, Faculty of Applied Sciences, University of Sri Jayewardenepura, Gangodawila, Nugegoda, Sri Lanka; ^2^Department of Food Science and Technology, Faculty of Livestock, Fisheries and Nutrition, Wayamba University of Sri Lanka, Makandura, Gonawila, Sri Lanka; ^3^Department of Horticulture and Landscape Gardening, Faculty of Agriculture and Plantation Management, Wayamba University of Sri Lanka, Makandura, Gonawila, Sri Lanka

## Abstract

Pasta is a widely consumed food in all over the world. Coarse semolina obtained from durum wheat and water are the main ingredients of conventional pasta products. The amount of gluten and quality level of durum wheat, are two important factors for the superiority of finished pasta. Market price of durum wheat is higher than the common wheat and it contributes no more than 5% of the world wheat production. Thus, to come across the challenge of emerging pasta consumption, new field of research that is dealing with the incorporation of nonconventional ingredients to the conventional formula of pasta has initiated. The compositions of raw materials which are used for pasta preparation directly affect the physical, chemical, and textural properties of the product. Therefore, incorporation of nonconventional ingredients can lead to a contradictory effect of pasta quality. This review will focus on the various types of nonconventional ingredients that are being incorporated in pasta products and their effect on the quality attributes of different pasta products.

## 1. Introduction

“Pasta” is an Italian word for “dough” [1]. The Italian style extruded foods namely spaghetti and lasagna are generally termed as pasta. It is a primeval food which is defined as a type of dough extruded or stamped into many shapes for cooking [2]. The world pasta production amounts to approximately 14 million tons in 2014 [3]. Traditionally, Italy is the main producer and leader of the pasta consumption in the world [1]. Pasta is far and widely consumed in the globe for the reason that of its convenience, palatability, and the longer shelf life than other bakery products, such as breads and buns [4].

Durum wheat (*Triticum durum*), which is the hardest wheat, is traditionally used to make pasta. Semolina, coarse particles produced by the milling process of durum wheat, is ideal for making pasta [1]. It provides pasta a good cooking quality and eating features will be better.

**Figure 1:**
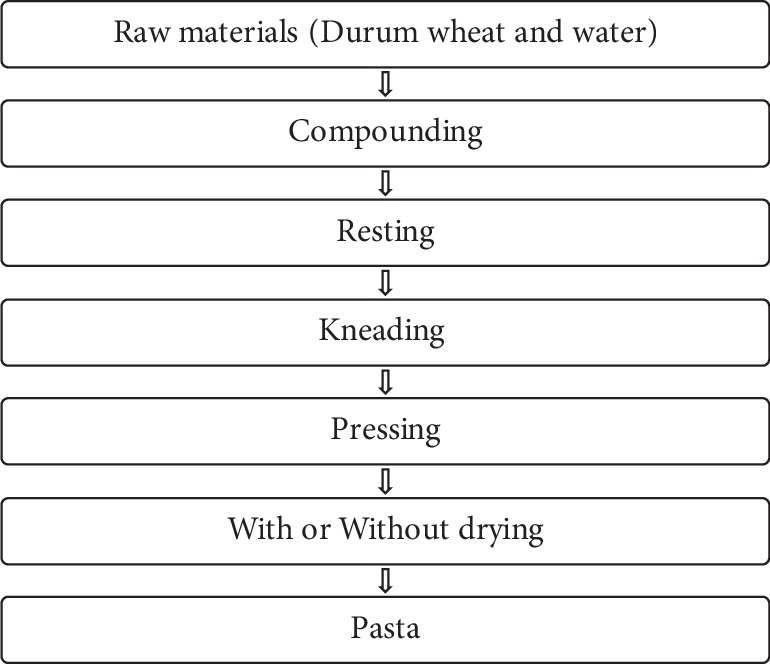
Conventional durum wheat pasta production process [8].

Figure 1 shows the conventional method of durum wheat pasta production. The important factors for the superiority of finished pasta are gluten content and quality of durum wheat. The pasta cooking quality and gluten strength are affected by the molecular weight of glutenin. Among high molecular weight glutenin subunits, (HMWGS) HMWGS 6 + 8 or 7 + 8 give better quality than HMWGS 20. Among low molecular weight glutenin subunits, (LMWGS) LMWGS 2 gives better quality than LMWGS 1 [5].

Relatively high yellow pigment content, low lipoxygenase activity and high protein content are important properties of durum wheat for good cooking quality of pasta [6]. However, Bustos et al. [4] have reported that wheat flour from both *Triticum durum* and *Triticum aestivum* can be utilized for pasta development and the protein content of durum wheat (on average 11.81% dry weight of defatted flour) is higher than the protein content of bread wheat (on average 11.08% dry weight of defatted flour). Also the protein content of bread wheat has a higher variation (13.54–5.71%) than durum wheat. Moreover the total content of gliadin and glutenin of bread wheat (61.53%) is not significantly different from the total content of gliadin and glutenin of durum wheat (61.42%) [7].

The carotenoids content of durum wheat is higher than that of bread wheat and therefore, pasta made with durum wheat shows more yellow color than bread wheat pasta [4]. Moreover, durum wheat pasta is dense and firm in texture and shows more elastic properties.

Relatively low in fat and sodium levels give a reason to consider pasta as a healthy food [9].

Its ease of use, low glycemic index and long shelf life have lead to become pasta as an interest of researchers [10]. In addition to that, pasta is a trendy food with a wide range of acceptability in many population groups including fitness enthusiasts [11].

Increased demand by growing number of health conscious consumers for healthy foods has shifted the interest of researchers and food manufacturers to develop pasta products rich in minerals, vitamins, fiber, and with low glycemic index. Bustos et al. [4] have reported that among the functional foods, pasta is an ideal vehicle for the wellbeing advancement depending on its low cost, long shelf life and high worldwide consumption. The World Health Organization (WHO) and Food and Drug Administration (FDA) consider pasta as an appropriate vehicle for the incorporation of nutrition supplements [12].

In the recent past, the food industry has made a continuous effort to introduce newer functional pasta products enriched with nutrients and bioactive compounds [13]. Accordingly, different grains have been utilized to substitute wheat semolina [14]. Incorporation of other grains has resulted in higher dietary benefits, such as increased essential amino acids, minerals, vitamins and phenolic compounds. In addition, for unique sustenance composite flours have been used to develop gluten-free or low glycemic index pasta. A few researches have examined the potential use of functional ingredients to produce pasta which are enriched with dietary fiber [15], bran [16], legume flour [17], whey and egg white powder [18], millet [19] and other plant materials [4, 19]. These studies demonstrate the increasing interest of consumers to use pasta as a functional food. Addition of beneficial ingredients to pasta can reduce the glycemic index and provides additional health benefits to consumers. Durum wheat, that provides the primary ingredient for pasta production namely semolina contributes only 5% to the total world wheat production and it is generally sold at a higher price than the common wheat [20]. Therefore, process development and product formulations to produce pasta from nonconventional ingredients are necessary to meet the demand of increasing pasta consumption [20]. This paper provides a brief overview on the use of nonconventional ingredients in pasta production.

## 2. Role of Starch and Gluten Protein on the Quality of Pasta

The compositions of raw materials which are used for pasta preparation directly affect the physical, chemical and textural properties of pasta [21]. Cooking quality is considered as the most important quality characteristics of pasta. Cooking time, cooking loss, water absorption index, swelling index, and texture are the parameters that determine the cooking quality [22]. Characteristics of semolina and the Maillard reactions that occur in food processing affect technological and sensory properties of pasta [23].

Starch network and the gluten protein of pasta products determine the quality of pasta [24]. They are related to starch composition and protein content of the pasta ingredients [25]. Glutenin and gliadin are the two main components of gluten.

When exposed to water glutenin and gliadin form a strong gluten network which is typical for wheat flour. This gluten network forms uniform and compact system with swelled starch granules during the cooking. Physical competition between protein coagulation and starch swelling determine the cooking quality and textural characteristics of pasta [26]. In the event that the protein coagulation wins, starch particles are caught in the system alveoli advancing firmness of cooked pasta. On the off chance that the starch swelling wins, the protein coagulates in discrete masses, coming up short on a constant system, and pasta will demonstrate nonabrasiveness and typically stickiness [9]. The associations between protein system development and starch gelatinization within the sight of water are identified with various textures and cooking characteristics of pasta [27].

High resistance to breakage and cooking tolerance, which are given by a strong gluten network are important parameters for low cooking losses and high water absorption of pasta products. During the cooking, coagulation of the gluten network reduces the elasticity and compactness of the protein and starch system of pasta products. Therefore the cooking loss is high due to the easy swelling of starch granules during cooking. The protein quality and content of the raw material determine the level of gluten content and more than 11–16% (dry weight basis) of protein in durum wheat is not suitable for dough formation as it is difficult to handle during the processing [8].

## 3. Purpose of Adding Nonconventional Ingredients to Pasta Products

During the most recent century, processed food sector has been changed according to the consumer preferences for healthy foods. Numerous improvements in the food industry and high consumer demand for the pasta products have directed the development of pasta products with nonconventional ingredients [22]. Incorporation of nonconventional ingredients such as dietary fiber, vitamins, minerals, natural pigments and antioxidants to pasta products improves the functional properties of conventional pasta products. The commonly used functional ingredients in pasta products and their sources are presented in Table 1.

During the flour processing most of the nutrients such as essential amino acids, minerals, and vitamins are removed from the wheat grains. As a result, generally wheat flour is rich in carbohydrates, than other nutrients [47]. Therefore, nonconventional ingredients have been added to pasta products as nutrient enhancers to enrichment or fortification. The lacking nutrients in pasta products and their sources are presented in Table 2.

## 4. Production of Pasta with Nonconventional Ingredients

Pasta is a reasonable vehicle for the consolidation of supplements such as plant extracts, vitamins, minerals, fatty acids and dietary fiber [56]. Researchers have investigated the potential of fruit waste [36], vegetables [61–63], cereals [64], fruit extracts [65], and legumes [34, 66] as nonconventional ingredients in pasta products.

## 5. Dietary Fiber Supplemented Pasta

Among the bioactive compounds dietary fiber represents a notable role [67] and it is an influential component in a healthy diet. Enrichment of foods with dietary fiber improves the functional properties of the food but leads to problems in technical quality [19].

For an example, composite flour with durum wheat semolina and dietary fiber can reduce the consumer acceptance because of low palatability. Biernacka et al. [28] have carried out a study to develop a pasta product enriched with carob fiber (CF) which is an insoluble and nonfermentable dietary fiber type. Results have showed that addition of carob fiber has a strong effect on color and has increased water absorption and optimal cooking time of pasta. Cappa and Alamprese [29] have studied production of fresh egg pasta which has been enriched by using brewer's spent grains (BSG), the main by-product in brewing industry. Addition of BSG has significantly lowered the thickness of the dough before and after cooking because of the low elasticity of the dough.

Moreover, addition of fiber reduces the average break strain of pasta products than conventional pasta products. However, additions of dietary fiber [35] and legumes [34] both have significantly increased the swelling index of pasta.

Whole grain flour types contain more dietary fiber and other bioactive compounds than refined flour types. Ciccoritti et al. [67] have explored the use of debranning products in pasta production in order to enhance the dietary fiber content and bioactive compounds. Pasta which has been developed with bran fractions and debranned kernels has displayed higher contents of total dietary fiber, arabinoxylans, protein and ash compared to conventional pasta with adequate scope of cooking characteristics. Foschia et al. [9] have revealed that incorporation of various dietary fiber into pasta cause a huge increment in water absorption than noted for conventional pasta. Expanded level of starch gelatinization and interruption of the protein starch network are the major reasons for the increased water absorption of pasta [22, 30, 68]. Fogliano and Vitaglione [69] have investigated the insulin enriched pasta. It is demonstrated that due to the protein and fiber interaction as well as protein and starch competition for water absorption, the structure of pasta has become less elastic and compact.

La Gatta et al. [68] have revealed that the loss of solids from whole-meal/semi-whole-meal pasta during cooking is occurred due to the uneven distribution of water and destruction of protein starch network in the pasta structure, which is resulted from the interference of fiber with the gluten protein matrix of pasta.

Kaur et al. [16] also have explored the potentiality of using several types of cereal bran namely wheat, oat, barley and rice for the development of fiber incorporated pasta. Increment in bran fraction from 5% to 25% has prompted to a huge increase in the murkiness of the product. Physical disturbance of the gluten network by the germ particles and bran which, gives a way of water retention in the pasta, leads to reduce the optimum cooking time of cereal bran incorporated pasta compared to conventional pasta. These outcomes have reasoned that cereal bran can be added up to 15% without unfavorably influencing the physicochemical, cooking and sensory qualities of pasta.

The utilization of different ingredients in pasta can give fascinating quality attributes, giving modifications in the dietary nature of the various formulations. The fibrous matter of the plant and starch which cannot be extracted during processing is called as bagasse. Fiorda et al. [70] have studied incorporation of cassava bagasse into pasta. Results have revealed that pasta firmness can be increased due to pregelatinized flour prevailed with high water retention which is caused by high fiber content from the cassava bagasse.

A summary of the effect of dietary fiber supplementation on pasta quality parameters is presented in Table 3.

## 6. Protein Supplemented Pasta

Few investigations have concentrated on expanding dietary benefits of pasta in terms of protein content [2, 20]. Cooking loss is an important parameter to measure the quality of pasta products. Low cooking loss is identified as the high quality of pasta [21]. The capability of gluten-starch network to retain the physical uprightness of pasta during cooking is responsible for the cooking loss. Protein network of pasta can be strengthened by thermal protein denaturation to improve the firmness of cooked pasta. As indicated by the Cappa and Alamprese [29] the mechanical properties of cooked pasta can be improved by incorporation of egg white powder because of the strong protein matrix created by ovalbumin and the break load and strain of cooked pasta can be increased more significantly, than in pasta without egg white powder.

Correia et al. [41] have investigated the effect of mushroom powder as a protein supplement in fresh pasta development. According to the results solid loss in the cooking water has been within the recommended level which is 9% [41]. Increments of mushroom powder content and drying temperature has led to reduce the adhesiveness, internal, and external firmness of pasta. Desai et al. [42] have studied the incorporation of fish (*Pseudophycis bachus*) powder on the physiochemical attributes of pasta. It is demonstrated that incorporation of fish powder can improve the protein, lipid and ash content of pasta while reducing the moisture and carbohydrate contents.

Protein interactions in a constant matrix as well as the content and quality of protein are imperative requirements to develop an ideal network of protein and carbohydrate to improve the cooking quality of pasta [71]. Desai et al. [42] have revealed that incorporation of fish powder can decrease the cooking quality because of disruption and weakening of the gluten protein network. Similar results have been reported by Ramya et al. [43] who have considered the incorporation of shrimp meat powder in pasta products and have detailed that the cooking loss (leaching of solids) is increased when there is increase in the incorporated amount of shrimp meat powder. Due to the higher cooking loss and lower water absorption the optimum cooking time is decreased when the shrimp meat powder is added.

As mentioned in the above sections, the swelling index of pasta is dependent on the competition between the starch and protein for water absorption. Swelling index of pasta is reduced with the addition of protein into the pasta formula, due to the formation of strong protein network which can reduce the water delivery for gelatinization and swelling of starch granules [42, 72]. Similar studies have been carried out by Ramya et al. [43], Desai et al. [42] and Yousif et al. [73] and they have demonstrated that lowering of starch swelling and water absorption of pasta have happened because of the competition of protein powder with the starch for water. Above findings are deviated from the finding of Devi et al. [46] as the incorporation of fish mince has increased the water absorption of pasta. This might be because of the strong protein-starch matrix formed by fish mince that have higher limits to absorb and hold water.

Firmness is an impression of the bond quality and the uprightness of the protein network in cooked pasta. Addition of fish powder [42], shrimp meat powder [74], and beef meat [72] has increased the firmness value of pasta. These results might be happening due to the low water absorption and low swelling index. Firmness can be reduced due to the higher swelling index and water absorption in pasta [9].

Among the animal protein sources, fish protein concentrate has been subjected to several researches because of its nourishing qualities. Goes et al. [52] have studied the fresh pasta enrichment with protein concentrate of tilapia. Results have revealed that incorporation of fish concentrate can increase the mineral profile, lipid content and protein content and reduce the caloric value of the pasta products.

In addition to the animal protein sources plant proteins also can be added to pasta products in order to improve its protein quality. Petitot et al. [44] and de la Peña et al. [45] have researched related to the incorporation of flour from bean varieties as sources of protein in pasta. The findings have demonstrated that incorporation of bean flour leads to reduce the cooking time of pasta. Laleg et al. [54] have researched on the effect of incorporation of legume flour in varying quantities on the structure, nutritional and sensory characteristics of pasta products. Results revealed that addition of legume protein increases the nutritional composition of pasta. Findings of Laleg et al. [75] have revealed that legume and wheat flour mixed pasta can improve the essential amino acid profile compared to conventional pasta products or egg enriched pasta products. In addition to that, Laleg et al. [54] have demonstrated that in vitro digestion of protein can be increased due to the weak protein network. A summary of the effect of protein supplementation on pasta quality parameters is presented in Table 4.

## 7. Antioxidants Supplemented Pasta

During the recent years consumer purchasing decision is based on the health benefits of the foods than the nutritional benefits. Antioxidants are one of the most important categories of bioactive compounds, which can assure the prevention of risk of the chronic inflammations. Some cereals are considered as antioxidants (i.e., polyphenols) rich food sources as they have potential to reduce the risk of noncommunicable diseases [76]. According to the investigations of Ciccoritti et al. [67] the level of antioxidants in pasta can be increased than conventional pasta by incorporating bran fraction and entire kernel of durum wheat to pasta products. Incorporation of entire kernel can be led to high preservation of phenolic compounds. During the cooking, bound phenolics can be extracted from the food matrix by the action of boiling water [77]. Therefore the bran fraction incorporated cooked pasta may have higher phenolics content than the conventional pasta.

Fares et al. [78] have investigated the addition of *β* glucan from barley and Bacillus coagulans BC30 into pasta products which has been developed with phenolics rich durum wheat flour. Results have revealed that careful milling process to protect aleurone layer of durum wheat grain can have a significant effect on the phenolics content of pasta.

Agro industrial by products are major sources that give antioxidants to the value added products. Apples are known as a good source of phenolic compounds [79], especially the peel [80]. According to the investigations of Lončarić et al. [39] incorporation of apple peel powder in pasta has increased the content of total polyphenols content and antioxidant activity significantly than in conventional pasta. However, it has increased the cooking loss and water absorption and has decreased the adhesiveness, hardness and sensory qualities of pasta.

Pasqualone et al. [80, 81] have carried out a study to investigate the potentiality of bran aqueous extract and bran oleoresin to produce functional pasta. Bran oleoresin incorporated pasta has shown significantly high hydrophilic and lipophilic antioxidant activities. De Paula et al. [82] have investigated the effect of processing on the physicochemical properties of *β* glucan, composition of phenolic acids and radical scavenging capacity of barley pasta. *β* glucans are bioactive compounds which are having antioxidant properties to reduce the risk of noncommunicable diseases [83]. Results have demonstrated that processing steps such as extrusion, drying, and cooking have considerable effect on *β* glucan physiochemical properties of flour, blends, and pasta products. In the case of free radical scavenging capacity and the content of total phenolics of barley pasta, there is no significant effect on them during the pasta processing [82]. In view of this, distinctive handling advances have been found to deliver different impacts on phenolics content, and accordingly the decision of cereal formula and technological process is significant in protecting phenolic acids and their health improving properties.

## 8. Pasta Products with Added Starch

Starch is a polymeric carbohydrate comprising of countless units of glucose joined by glycosidic bonds and it is the major energy storage of green plants. In pasta production starch is added to improve the appearance, surface smoothness, and mouth feel of the final product.

According to the findings of Fiorda et al. [70] greater stickiness can be observed due to the high amylopectin content which influences the texture of the products after adding starch to the pasta formulations. A preliminary study conducted by Ibitoye et al. [84] has investigated sweet potato starch and wheat flour blended noodles. Results have demonstrated that there is no any significant difference of overall acceptability between noodles which contained 30% potato starch and conventional formula. Menon et al. [85] have studied the effect of various sources of starch such as banana, lentil, black gram and sweet potato on pasta quality. Among the starches, highest cooking loss has been observed in pasta fortified with 10% lentil starch. Crude protein contents have been higher for black gram and sweet potato starch-fortified pasta samples. Firmness of cooked pasta has been higher for black gram starch fortification, while highest toughness has been recorded for lentil starch based cooked samples.

## 9. Pasta Production with Composite Flour

Wheat flour which is mixed with other flour for different purposes called as composite flour. Legumes, yam, maize, soy bean, sweet potato, and cassava flour are widely used to prepare composite flour. Several researchers have studied the use of composite flour in pasta making.

The micronization is a process that reduces the particle size of ingredients to increase the solubility or bioavailability of particles. Junqueira et al. [86] have evaluated the impact of using wheat semolina and micronized corn pericarp in spaghetti type pasta. It has significantly contributed to the protein, ash, carbohydrate, and cooking loss of pasta. However, the moisture content, lipid content, caloric value, cooking time, weight gain, and volume increase parameters have not been significantly affected.

Bouasla et al. [87] have carried out a study to develop pre-cooked rice pasta enriched with legume flours (yellow pea, chick pea and lentil) in order to produce gluten free spaghetti type pasta product. Legumes are rich source of proteins, fibers, vitamins, and minerals when compared with other flour types. According to the findings, incorporation of legume flour decreases expansion ratio and lightness, and increases yellowness, firmness, and adhesiveness, without affecting the minimal preparation time. Incorporation of amaranth flour in processing of pasta has produced a low level of stickiness that is more desirable for the pasta products [70].

Ginting and Yulifianti [88] have studied characteristics of noodle prepared from orange-fleshed sweet potato and bread wheat flour. The results have suggested that starch characteristics such as swelling power, pasting properties and amylose-amylopectin ratio are affected to the noodle texture other than the gluten protein content of wheat flour.

Low glycemic index is an important factor of a diet for both healthy and diabetic subjects. Generally, seeds of legumes give moderate postprandial blood glucose increase [89] and Goñi and Valentín-Gamazo [11] have observed that the addition of chickpea flour decreases the glycemic response of pasta in healthy people. Results have demonstrated that combination of wheat and chickpea enhances the level of quality protein in pasta products as legumes are rich in lysine which is a limiting amino acid in cereal and cereals are rich in methionine which is a limiting amino acid in legumes. Pasta products which contained chickpea flour have presented a low glycemic response. This can help widen the scope of low GI foods accessible to the consumer.

Gull et al. [90] have studied the optimization and functionality of millet supplemented pasta. According to the results millet flour cause significant increase in cooking loss because of the low gluten level results in poor protein network. According to the findings of Cárdenas-Hernández et al. [14] incorporation of flour from dried amaranth leaves and amaranth seed decreased the cooking time and increased the cooking loss percentage. Peanut flour is low fat, high protein functional ingredient which is prepared from defatted roasted peanut kernels. Howard and Hung [91] have formulated pasta with peanut flour. Potential positive attributes of peanut pasta include its unique flavor, whereas a potential negative attribute seems to be its softer texture upon cooking.

Lorusso et al. [92] have investigated the use of fermented quinoa flour for pasta making.

Quinoa is a pseudo-cereal which has a high-protein content (14–16 g/100 g) [93]. Its amino acid composition, and high amount of histidine and lysine, are near to the perfect protein balance recommended by the FAO [12]. Addition of 20% (w/w) of quinoa flour to semolina has been productive in enhancing the dietary attributes without affecting the sensory and technological quality of pasta. Moreover, the study has revealed that fermentation of quinoa flour with lactic acid bacteria can additionally improve the beneficial outcomes of quinoa. As indicated by this research it can be concluded that the nutritional potential of pasta can be successfully improved through the fermentation technology of ingredients. This is reasonable to be incorporated into the future food propensities improvement. The commonly used potential sources for composite flour in pasta production are presented in Table 5.

## 10. Gluten Free Pasta Products

Minimum cooking loss, good texture, minimum surface stickiness and resistance to surface disintegration are some of the important characteristics which are given by gluten protein in durum wheat semolina.

Celiac disease is a typical digestive system related condition where an individual has an unfavorable response to gluten. The gliadin content in gluten protein is responsible for this condition [94]. Therefore, development of gluten free diets is the most ideal way to avoid this type of diseases. As the gluten protein plays an important role to improve the cooking quality of pasta, careful attention should be given to the development of gluten free pasta products. Selection of suitable formulations with appropriate amounts of protein, hydrocolloids and moisture to achieve the desirable quality can replace the role of gluten in pasta [95].

At present, the most commonly used ingredients in the development of gluten free pasta are flour from cereals such as rice, corn and buckwheat [96, 97], pseudocereals [12, 70] starches [98], dairy and vegetable proteins [99].

One of the typical procedures to develop gluten free pasta is to acquire pre gelatinized starch through cool and heat stages, in this way a rigid network is formed depending on the retrograded starch [100]. Larrosa et al. [101] have investigated the effects of egg proteins in gluten free pasta. Corn flour has been used as the main raw material. Results have revealed that egg protein can be used as a protein source in gluten free pasta development. Menga et al. [102] have developed gluten free pasta with chia and rice flour. Chia is a species of flowering plant in the mint family. Hydrocolloids are good alternates which can imitate the role of gluten network in gluten free pasta.

Protein level is essential for good cooking properties of pasta and for nutritional value. Phongthai et al. [18] have investigated the rice flour based gluten free pasta. Different protein concentrate including egg albumen, rice bran protein, whey protein, and soy protein have been used to enrich the rice flour based gluten free pasta in order to improve the cooking quality. According to the results it has been concluded that the soy protein and egg albumen can be used as sources of protein in gluten free pasta as they do not significantly affect the cooking quality of pasta.

## 11. Conclusion

Pasta is a staple food in many countries all over the world. Though pasta is simple and easy to produce, the cost of main ingredient durum wheat semolina is significant in popularizing pasta. Many research studies have been conducted around the world to develop pasta products with nonconventional ingredients and added functional properties to meet the demand of health conscious consumers.

The compositions of raw materials which are used for the pasta preparation directly affect the physical, chemical and textural properties. Therefore, incorporation of nonconventional ingredients leading to contradictory effect of pasta quality and incorporation of nonconventional ingredients without affecting the quality attributes of pasta is somewhat difficult. Thus, more attention is required to innovate novel nonconventional ingredients to improve the quality of the dough and to develop nutritious pasta products with better quality attributes. In this way, more research is required to recognize and develop nonconventional ingredients with better usefulness and sensible expenses to produce novel pasta products.

## 12. Future Research Trends

Among healthy foods, pasta is an ideal food for wellbeing advancement depending on its high global consumption. Though pasta can be fortified, enriched or supplemented with nonconventional ingredients, there should be a greater attention to the cooking quality of the pasta product. Research studies can be focused on the interactions between nonconventional ingredients and the dough matrix, individually in order to improve the quality of the final product with the nutritional or functional properties.

## Figures and Tables

**Table 1:** Sources of functional ingredients in pasta products.

Functional ingredient	Potential supplying sources	References
Dietary fiber	Carob fiber, brewer's spent grain, legume flour, orange by product fiber	[28–37]
Natural pigments	Anthocyanins, betalains, carotenoids	[2, 38]
Antioxidants	Apple peel powder, carrot powder, grape powder	[2, 39, 40]
High biological value protein	Egg white powder, mushroom powder, fish protein powder, shrimp meat powder, bean flour and soy flour, fish mince, yeast protein concentrate	[29, 30, 41–46]

**Table 2:** Sources of nutrients in pasta products.

Nutrient	Potential supplying sources	References
Vitamins	Vegetables, calf liver, germinated plant seeds, seaweeds	[48–50]
Minerals	Selenium enriched durum wheat, fish concentrate, cereal bran, germ	[51–53]
Essential amino acids	Fava protein, chick peas, quinoa flour, common bean flour, milk and milk products, whey protein, cottonseed meal	[11, 12, 30, 54, 55]
Polyunsaturated fatty acids	Long chain n-3 polyunsaturated fatty acids	[56–58]
Essential oils	Thymol, menthol	[59, 60]

**Table 3:** The effect of dietary fiber supplementation on pasta quality parameters.

Quality parameter	Effect	References
Water absorption	Increase	[28, 30, 35]
Thickness	Decrease	[29]
Average break strain point	Decrease	[29]
Swelling index	Increase	[30, 31, 33–35]
Elasticity	Decrease	[69]
Optimal cooking time	Increase or decrease	[16, 28]

**Table 4:** The effect of protein supplementation on pasta quality parameters.

Quality parameter	Effect	References
Average break strain point	Increase	[29]
Internal and external firmness	Decrease	[41]
Adhesiveness	Decrease	[41]
Cooking loss	Increase	[42, 43, 54]
Swelling index	Decrease	[42, 72]
Water absorption	Decrease	[42, 43]
Firmness	Increase	[42, 43, 46, 72]
Optimum cooking time	Decrease	[44, 45]

**Table 5:** Potential sources for composite flour in pasta production.

Source of flour	References
Orange fleshed sweet potato flour	[88]
Legumes	[11, 87]
Amaranth flour	[14, 70]
Finger millet flour	[19]
Peanut flour	[91]
Quinoa flour	[92]
